# Comparison of energy expenditure with level of amputation in patients with diabetes mellitus

**DOI:** 10.1111/wrr.70007

**Published:** 2025-02-23

**Authors:** Jan Karel Petric, Matthew J. Johnson, Kelly Jeans, Ariel R. G. Fortenberry, Jijia Wang, Kirsten Tulchin‐Francis, Dane K. Wukich, Tiffany D. F. Graham

**Affiliations:** ^1^ University of Texas Southwestern Medical Center Dallas Texas USA; ^2^ Scottish Rite for Children Dallas Texas USA; ^3^ Nationwide Children's Hospital Columbus Ohio USA

**Keywords:** 6MWT, diabetes, energy expenditure, FAAM, level of amputation, metabolic cost, PROMIS‐29, prosthetics, transmetatarsal, transtibial

## Abstract

Diabetes mellitus (DM) is the leading cause of non‐traumatic lower extremity amputations in the USA. After these amputations, patients exhibit reduced mobility and increased energy demands of walking. The best surgical practice is to preserve as much of a functional limb as possible, in part due to the fact that proximal amputations result in a greater increase in energy expenditure compared to more distal amputations. While differences in transfemoral, transtibial and partial foot amputation levels have been previously documented, no studies have directly compared transtibial and transmetatarsal amputees. The present study aimed to compare energy expenditure and patient‐reported outcomes in patients with diabetes mellitus who have undergone transmetatrsal (TMA) and transtibial amputations (TTA). Thirty‐nine DM participants with either unilateral TMA, unilateral TTA or no amputations (control group) participated in this observational study. Energy expenditure, heart rate (HR) and distance travelled during six‐minute walk test (6MWT), the Foot and Ankle Ability Measure (FAAM) and the Patient‐Reported Outcomes Measurement Information System (PROMIS‐29) were measured at a single research visit. No significant differences between the three groups were detected in energy expenditure, HR or distance covered during 6MWT, as well as in PROMIS‐29 or FAAM patient‐reported outcomes. While the results of this study suggest no differences in functional and patient‐reported outcomes between transmetatarsal and transtibial amputees, a larger sample size that would allow for control of comorbidities is needed.

**Abbreviations**
6MWT: six‐minute walk test
ADL: activities of daily living
DM: diabetes mellitus
FAAM: Foot and Ankle Ability Measure
HRQOL: health‐related quality of life
LEA: lower extremity amputation
LEFS: lower extremity functional scales
NRS: numerical rating scale
SF‐36: Short Form 36
TFA: transfemoral amputees
TMA: transmetatarsal
TTA: transtibial
VAS: visual analogue scale

## 1 INTRODUCTION

Diabetes is the leading cause of non‐traumatic lower extremity amputation (LEA) in the USA.^1^ Individuals who undergo LEA are at risk for reduced mobility, impaired cardiac reserve and subsequently worse quality of life.^2^ In people with diabetes mellitus (DM), walking at baseline requires increased energy compared to those without diabetes.^3^, ^4^ Once a patient with DM undergoes LEA, the increased energy demand and reduced mobility can be debilitating, leading to a higher incidence of mortality.^5^, ^6^, ^7^

The goal of amputation is to preserve functional limb length, by amputating at the most distal level possible. Higher relative energy costs have been reported in transfemoral amputees (TFA) when compared to transtibial (TTA)^8^, ^9^ and Syme's,^8^ while Syme's and midfoot amputations were not significantly different when compared with TTA.^8^, ^9^ Different studies found no difference in energy expenditure between TFA, TTA and partial foot amputation.^10^ However, the partial foot amputation group in their study included only patients with amputation at the midfoot or proximal, and no patients with transmetatarsal (TMA) were included.

While a foot salvage amputation may offer advantages over TTA, from an energy cost standpoint, the literature has not demonstrated a clear advantage of performing a partial foot amputation over a TTA. Patients who undergo amputation at or more proximal to the midfoot/Lisfranc joint level lose muscle attachments of the peroneus longus and brevis, tibialis anterior and portions of the tibialis posterior muscle. Without maintenance of musculotendinous insertion, plantarflexion, dorsiflexion, supination and pronation can be impacted resulting in abnormal gait patterns. TMA is generally preferred over TTA, as the patient can walk without a prosthesis, key muscle attachments are preserved, and the ankle joint remains functional. To the best of our knowledge, no prior study has directly compared TMA to TTA with regard to energy expenditure. The purpose of this study is to compare energy expenditure in patients with diabetes mellitus who have undergone TMA and compare those patients who have undergone TTA and returned to ambulation with a prosthesis. Both groups were also compared to the control group of patients with diabetes mellitus who do not have any LEA. We will also compare patient self‐reported outcomes to assess the impact of these LEAs on quality of life.

## 2 PATIENTS/MATERIALS AND METHODS

This clinical study was conducted from February to August 2022. All participants signed an informed consent form prior to inclusion in the study. The work described has been carried out in accordance with the Declaration of Helsinki for experiments involving humans. Individuals who underwent TMA or TTA secondary to complications related to DM were eligible to participate. The control group consisted of participants with DM and with no prior LEA. Participants were included if they were over the age of 18 and did not have any open wounds or ulcers which would compromise their ability to walk for 6 min. All participants were instructed to use their previously fitted orthotic or prosthetic devices. The University of Texas Southwestern Medical Center's (Dallas, Texas, USA) Prosthetic‐Orthotic clinic, as well as Orthopaedic Surgery clinic, served as the main recruitment sites. Patients were excluded if they had a contralateral TMA or major limb amputation; if they smoked, they were non‐ambulatory or were unable to provide informed consent.

### 2.1 Testing

Potential participants were invited to a single research visit and were asked to abstain from eating or drinking (other than water) for a minimum of 2 h prior to testing. A certified prosthetist–orthotist evaluated prosthetic fit and the absence of wounds. Patients were fit with the K5 oxygen analysis telemetry unit (COSMED, Chicago, IL, USA) and heart rate monitor (Garmin, Ltd., Olathe, KS, USA). Testing began with a seated rest period of 5 min, which served as a baseline. They completed a six‐minute walk test (6MWT) at a self‐selected speed to record the distance travelled in 6 min as well as to measure their oxygen consumption. The 6MWT was conducted in an enclosed corridor, on a course that included two cones 15 m apart for a lap of 30 m total. Participants were permitted to use a gait aid that they would normally use for such an activity and would allow them to safely complete the test, such as a cane or rolling walker. Participants were instructed to walk from one cone to the other, covering as much distance as possible in 6 min. After 6 min had elapsed, participants were asked to stop walking, and the distance was measured and recorded.^11^ At this time, participants had a second seated rest period until their heart rate decreased to approximately baseline values. Participants then filled out validated patient‐reported outcomes instruments: the Foot and Ankle Ability Measure (FAAM) and the Patient‐Reported Outcomes Measurement Information System (PROMIS‐29). FAAM is a validated measurement of physical function in individuals with a broad range of musculoskeletal disorders of the leg, foot and ankle and consists of two subscales: activities of daily living (ADL) and Sports.^12^, ^13^ PROMIS‐29 measures pain intensity and seven health domains (physical function, fatigue, pain interference, depressive symptoms, anxiety, ability to participate in social roles and activities and sleep disturbance). Specifically, the physical and mental health summary scores for PROMIS‐29 were used for analysis.^14^

### 2.2 Data and statistical analysis

Statistical analyses were carried out using SAS 9.4 (SAS Inc., Cary, NC, USA). Descriptive statistics were employed for summarizing numerical and categorical variables. Fisher's exact test was used to compare the categorical variables between groups. ANOVA with Tukey's HSD test and Kruskal–Wallis test was used to compare the numerical variables between three groups, as appropriate. A two‐sample t test was used to compare the time since amputation between TMA and TTA groups. The level of significance was set at 5%.

Data reduction for oxygen consumption testing collected during the 6MWT included: 1 min of steady‐state data during the pretest period (e.g., 3:30–4:30) and during the 6MWT (e.g., 9:30–10:30) was selected for analysis.^15^ Primary outcome variables included resting VO_2_ rate (mL/kg/min), resting heart rate (bpm), walking VO_2_ cost (mL/kg/m), walking heart rate (bpm) and self‐selected walking velocity (m/min).^16^

## 3 RESULTS

### 3.1 Demographics

**FIGURE 1:**
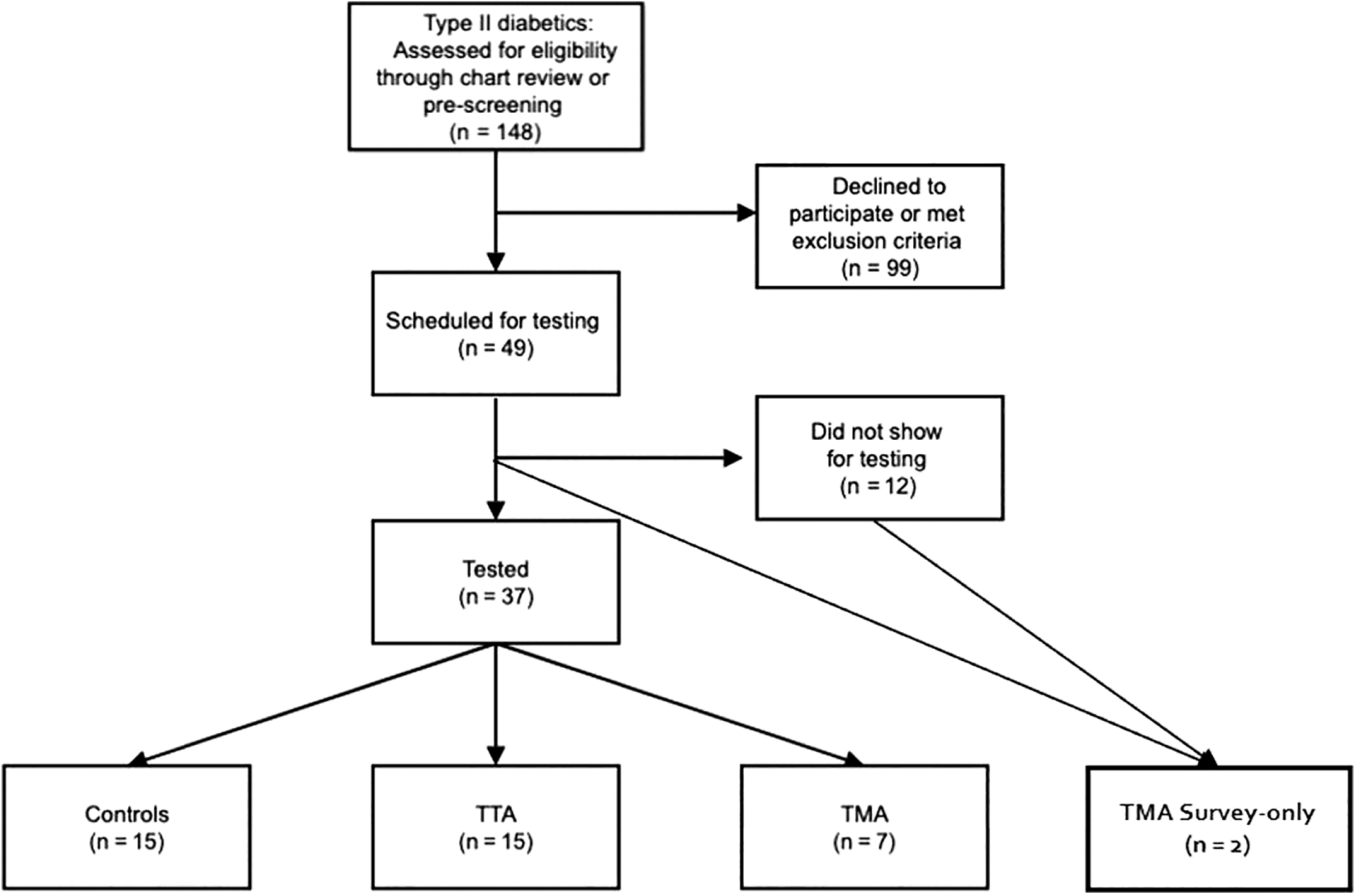
Flow chart of the participant recruitment. TMA, transmetatarsal amputation; TTA, transtibial amputation.

Thirty‐nine participants enrolled in the study (Figure 1). Fifteen participants in both the TTA and control groups completed a full protocol (6MWT and PROs). The TMA group had a total of nine participants. Out of nine participants, seven completed the full protocol, and two participants only completed PROs. All 15 participants in the TTA and control groups were used for analyses of the 6MWT and PROs. In the TMA group, seven participants were used in the 6MWT and PRO analyses (for a combined 37 participants) and all nine were used in PRO analyses (total of 39 participants). Participant's characteristics at the time of consent, including time since amputation, are presented in Table 1. No between‐group differences in age or gender distribution were observed in full protocol participants (*p* = 0.1135 and 0.5063, respectively) as well as in PRO participants (*p* = 0.1061 and 0.3990, respectively). Additionally, no differences (*p* = 0.1930) in time since amputation were observed between the TTA (7.03 ± 2.96 years) and TMA (4.27 ± 4.97 years) groups.

**TABLE 1:** Demographic characteristics of the patients.

	TMA		TTA	Control	*p*‐value
Characteristics	Full protocol (*n* = 7)	Total survey (*n* = 9)	(*n* = 15)	(*n* = 15)	
Gender, male/female	6/1	8/1	13/2	10/5	0.5063
Age, mean (SD)	61 (11)	60 (10)	56 (7)	64 (10)	0.1135
Time since amputation, years (SD)	4.27 (4.97)		7.03 (2.96)		0.1930

Abbreviations: TMA, transmetatarsal amputation; TTA, transtibial amputation.

### 3.2 6MWT

In each of the three groups, participants elected to use gait aids during the testing (TTA: 1 rollator, 1 cane; CONTROL: 2 cane, 3 rollators; TMA: 1 rollator, 1 cane). Mean walking VO_2_ cost was 0.23 mL/kg/m (SD = 0.08), 0.22 mL/kg/m (SD = 0.05) and 0.21 mL/kg/m (SD = 0.07) for TMA, TTA and control groups, respectively. Mean distance walked during 6MWT in TMA, TTA and control groups was 313 m (SD = 74), 306 m (SD = 78) and 336 m (SD = 83), respectively. Mean walking HR was 100 bpm (SD = 12) for the TMA group, 103 bpm (SD = 16) for the TTA group and 99 bpm (SD = 18) for the control group. No significant differences were detected in walking VO_2_ costs between the three groups and between any pairwise group comparisons. Furthermore, no significant differences in distance covered and walking HR between the TMA, TTA and control groups were detected. Data are presented in Table 2.

**TABLE 2:** Results of over ground walking during six‐minute walk test and patient‐reported outcomes of participants, stratified by amputation level.

	TMA	TTA	Control	*p*‐values
Over ground walking				
VO_2_ cost, mean [mL/kg/m] (SD)	0.23 (0.08)	0.22 (0.05)	0.21 (0.07)	0.8656
6MWT distance, mean [m] (SD)	313 (74)	306 (78)	336 (83)	0.5909
HR, mean [BPM] (SD)	100 (12)	103 (16)	99 (18)	0.8457
PROs				
PROMIS				
Physical Health Summary, mean (SD)	45 (11)	44 (10)	46 (10)	0.8745
Mental Health Summary, mean (SD)	51 (10)	53 (8)	51 (9)	0.7657
FAAM				
ADL, mean (SD)	74 (23)	77 (13)	77 (19)	0.9019
Sport, mean (SD)	59 (35)	45 (28)	58 (28)	0.4003
Functional self‐score				0.3963
Severely abnormal, *n* (%)	1 (11)	0 (0)	0 (0)	
Abnormal, *n* (%)	2 (22)	5 (33)	5 (33)	
Nearly normal, *n* (%)	3 (33)	9 (60)	8 (53)	
Normal, *n* (%)	3 (33)	1 (7)	2 (13)	

Abbreviations: 6MWT, six‐minute walk test; ADL, activities of daily living; FAAM, Foot and Ankle Ability Measure; PROMIS, Patient‐Reported Outcomes Measurement Information System; TMA, transmetatarsal amputation; TTA, transtibial amputation.

### 3.3 Patient‐reported outcomes

Physical health summary scores were 45 (SD = 11), 44 (SD = 10) and 46 (SD = 10) for TMA, TTA and control groups, respectively. Mental health summary scores were 51 (SD = 10), 53 (SD = 8) and 51 (SD = 9) for TMA, TTA and control groups, respectively. Neither PROMIS‐29 physical nor mental health summary scores were significantly different among the three groups. Data are presented in Table 2.

For the FAAM, the ADL subscore was 74 (SD = 23), 77 (SD = 13) and 77 (SD = 19) for the TMA, TTA and control groups, respectively. In the Sport subscore, averages were 59 (SD = 35) in TMA, 45 (SD = 28) in TTA and 58 (SD = 28) in the control group. The answers (severely abnormal, abnormal, nearly normal and normal) in the functional self‐score section were not significantly different among the three groups. Furthermore, no significant differences were observed in ADL or Sport subscores. Data are presented in Table 2.

## 4 DISCUSSION

The purpose of the current study was to investigate energy expenditure, as well as self‐reported physical and mental outcomes in patients with diabetes mellitus without amputations (control) and patients who underwent unilateral transtibial or transmetatarsal amputations. There was no difference detected in walking VO_2_ cost, mean distance covered or mean HR during 6MWT. Furthermore, no significant differences in PROs were observed, which included PROMIS‐29 Physical Health and Mental Health scores, as well as ADL subscores, Sport subscores and functional self‐score in the FAAM questionnaire.

### 4.1 Energy expenditure

Prior studies have compared differences in physical performance between patients with different levels of amputation.^8^, ^9^, ^10^, ^17^, ^18^, ^19^, ^20^ Waters et al.^8^ reported higher energy consumption with higher amputation levels in 41 vascular and 29 traumatic amputees with either Syme's (amputation of the foot through the ankle joint), transtibial or transfemoral amputations. They concluded that vascular amputees have a higher energy consumption than traumatic amputees and that Syme's amputees expended the least energy during walking, followed by transtibial and transfemoral amputees. Gait velocity, which relates to distance travelled during 6MWT, was higher in Syme's compared to transtibial vascular amputees. However, they found no difference in HR between the two groups. Increase in oxygen consumption with higher levels of amputations was similarly reported in other studies.^9^, ^17^ Pinzur et al.^9^ reported an increase in energy demand with more proximal amputation levels; however, no difference in energy consumption was observed when comparing midfoot amputations with TTAs. Lin‐Chan et al.^18^ discussed a case report where a Syme's amputee underwent elective TTA and experienced 3%–5% lower energy consumption post‐TTA. Tekin et al.^19^ studied 10 transtibial unilateral traumatic amputees and nine limb salvage patients and reported no changes in distance travelled or energy consumption in 6MWT between TTA or limb salvage amputees. Göktepe et al.^10^ studied 64 unilateral traumatic amputees and reported no significant difference in energy expenditure between transfemoral, transtibial and partial foot amputees. While not statistically significant, comparing TTA and partial foot groups resulted in lower energy expenditure observed in the TTA group. In contrast to our study, the partial foot group in Goeptke et al. only included Chopart (amputation of the forefoot through the midtarsal joint), Pirogoff (amputation of the foot at the ankle, preserving a portion of the calcaneus to form a calcaneotibial arthrodesis) and Lisfranc amputations⸺all proximal to transmetatarsal amputation.^10^ Participants were also non‐diabetic, and the age range was 23–34 years.^10^ Similarly, the limb salvage population studied by Tekin et al.^19^ were non‐diabetic, traumatic amputees with an age range of 20–37 years.

Similar findings between the levels of amputation in our study may be explained by understanding how a prosthesis affects the biomechanics of human gait. The most efficient walking has been reported to be generated by the trailing leg's push‐off force.^21^ Additionally, a sufficient roll over an immobilized ankle does not increase energy expenditure.^22^ In a matched control group study by Dillon and Barker^23^ patients who underwent partial foot amputation (TMA, midfoot) and wore a prosthesis with AFO demonstrated relatively normal excursion of foot pressures that normalized knee and ankle moments. Their study did not include patients with TTA. Göktepe et al.^10^ compared partial foot and transtibial amputees in their study and found that ankle function and foot leverage are more restored by a transtibial prosthesis than a partial foot prosthesis. This suggests that transtibial prostheses provide a more symmetrical and energy‐efficient gait than partial foot prostheses,^10^ which may help explain how the transtibial amputees demonstrated similar levels of energy expenditure to TMA in our study, despite the higher level of amputation.

### 4.2 Patient‐reported outcomes

Patient‐reported outcomes (PROMIS‐29 and FAAM) have been studied previously in both the diabetic population and patients with various levels of amputations.^12^, ^24^, ^25^, ^26^, ^27^ However, our study is the first to compare diabetic TTA directly with TMA and diabetic control patients. Wukich et al.^27^ evaluated health‐related quality of life (HRQOL) and lower extremity function in 81 patients with diabetes mellitus who underwent TTA. HRQOL and lower extremity function improved significantly after TTA. They concluded that in patients with lower limb instability and/or chronic infections, TTA can significantly improve the quality of life and function of the lower extremities. However, they acknowledged that while 75% of patients improved their quality of life, 25% regressed. While no significant differences were found in the current study, results from Wukich et al.^27^ similarly suggest that TTA does not result in decreased quality of life, when compared to non‐amputated patients with diabetes mellitus. Deldar et al.^28^ compared functional and patient‐reported outcomes in patients who underwent TMA (*N* = 105) to those requiring more proximal lower limb amputation (*N* = 46; TTA = 18). They found that mean PROMIS‐29 pain interference and PROMIS‐29 pain intensity scores were not significantly different between higher‐level amputee patients and those with a successful TMA. However, when using the numerical rating scale (NRS) to directly assess pain levels in the past 24 h, they reported significantly greater acute pain levels in higher‐level amputees compared to TMA only. Using lower extremity functional scales (LEFS) to assess patient‐reported functional outcomes, no significant differences in functionality scores were observed. The cohort in this study was similar to ours, with more than 80% of participants having diabetes mellitus, with an average age of 61 years. Deldar et al.^28^ suggest that patients with TMA have a similar quality of life than patients with more proximal amputations, which current data support. While using the visual analogue scale (VAS) to measure pain and Short Form 36 (SF‐36) to assess quality of life, Tekin et al.^19^ reported higher quality of life (general health and vitality) and lower pain scores for the TTA group compared to limb salvage group. While this was a different population (traumatic, younger adults), it suggests, similarly to our study, that a higher amputation level (TTA) does not result in a decreased quality of life.

Limitations of the present study include the lack of control for patient comorbidities such as peripheral neuropathy or Charcot arthropathy that could affect walking expenditure. Further information regarding the pre‐amputation activity status of our participants would provide valuable information to the readers. However, the information was not found in their medical record or collected during study visits. Additionally, prosthetic componentry selection and residual limb length could affect energy efficiency and quality of life in the TTA group. The type of orthosis could also impact results for the TMA group. Some subjects used only foot orthoses, while others used ankle–foot orthoses, which could also impact gait kinematics. Since this was a pilot investigation, a formal priori power analysis was not conducted. The reader should consider these limitations when interpreting our results. To the best of our knowledge, this is the first study to investigate differences in energy expenditure and patient‐reported outcomes between TTA and TMA. The data from this study will provide information for a sample size for a further larger study.

## 5 CONCLUSION

The results of the current study suggest that there is no difference in energy consumption, 6MWT distance and HR during walking between transmetatarsal, transtibial and non‐amputee diabetic patients. The lack of performance differences may lead to similar patient‐reported outcomes between the groups studied. This information aids clinicians in counselling patients who have TMA and may be indicated to convert to BKA, while prior studies^8^, ^9^ have demonstrated a higher level of amputation increases energy expenditure and demand on the patient, and our study shows that this may not be the case. A larger sample size is needed to definitively determine whether there are no significant differences among the three groups.

## CONFLICT OF INTEREST STATEMENT

The authors declare no conflicts of interest.

## Data Availability

The data that support the findings of this study are available from the corresponding author upon reasonable request.
